# Machine Learning for the Early Prediction of Delayed Cerebral Ischemia in Patients With Subarachnoid Hemorrhage: Systematic Review and Meta-Analysis

**DOI:** 10.2196/54121

**Published:** 2025-01-20

**Authors:** Haofuzi Zhang, Peng Zou, Peng Luo, Xiaofan Jiang

**Affiliations:** 1 Department of Neurosurgery Xijing Hospital Fourth Military Medical University Xi'an China

**Keywords:** machine learning, subarachnoid hemorrhage, delayed cerebral ischemia, systematic review

## Abstract

**Background:**

Delayed cerebral ischemia (DCI) is a primary contributor to death after subarachnoid hemorrhage (SAH), with significant incidence. Therefore, early determination of the risk of DCI is an urgent need. Machine learning (ML) has received much attention in clinical practice. Recently, some studies have attempted to apply ML models for early noninvasive prediction of DCI. However, systematic evidence for its predictive accuracy is still lacking.

**Objective:**

The aim of this study was to synthesize the prediction accuracy of ML models for DCI to provide evidence for the development or updating of intelligent detection tools.

**Methods:**

PubMed, Cochrane, Embase, and Web of Science databases were systematically searched up to May 18, 2023. The risk of bias in the included studies was assessed using PROBAST (Prediction Model Risk of Bias Assessment Tool). During the analysis, we discussed the performance of different models in the training and validation sets.

**Results:**

We finally included 48 studies containing 16,294 patients with SAH and 71 ML models with logistic regression as the main model type. In the training set, the pooled concordance index (C index), sensitivity, and specificity of all the models were 0.786 (95% CI 0.737-0.835), 0.77 (95% CI 0.69-0.84), and 0.83 (95% CI 0.75-0.89), respectively, while those of the logistic regression models were 0.770 (95% CI 0.724-0.817), 0.75 (95% CI 0.67-0.82), and 0.71 (95% CI 0.63-0.78), respectively. In the validation set, the pooled C index, sensitivity, and specificity of all the models were 0.767 (95% CI 0.741-0.793), 0.66 (95% CI 0.53-0.77), and 0.78 (95% CI 0.71-0.84), respectively, while those of the logistic regression models were 0.757 (95% CI 0.715-0.800), 0.59 (95% CI 0.57-0.80), and 0.80 (95% CI 0.71-0.87), respectively.

**Conclusions:**

ML models appear to have relatively desirable power for early noninvasive prediction of DCI after SAH. However, enhancing the prediction sensitivity of these models is challenging. Therefore, efficient, noninvasive, or minimally invasive low-cost predictors should be further explored in future studies to improve the prediction accuracy of ML models.

**Trial Registration:**

PROSPERO (CRD42023438399); https://tinyurl.com/yfuuudde

## Introduction

Subarachnoid hemorrhage (SAH) is the third most common type of stroke and is usually associated with the rupture of an aneurysm [1]. The incidence of SAH is approximately 7.9 (95% CI 6.9-9.0) per 100,000 person-years globally, 8.3 (95% CI 7.2-9.5) per 100,000 person-years in Europe, 10.4 (95% CI 5.9-18.6) per 100,000 person-years in Asia, and 8.5 (95% CI 7.1-10.2) per 100,000 person-years in North America [2]. Recently, a discernible decline has been observed in global SAH incidence, which is potentially attributable to effective public health initiatives and the promotion of healthy lifestyles, especially effective management of certain modifiable risk factors (eg, reduction in smoking prevalence, management of hypertension) [3]. However, aneurysmal SAH continues to pose significant challenges, as evidenced by an in-hospital mortality rate of nearly 20% [4]. Of these, delayed cerebral ischemia (DCI) is the leading cause of death, with a prevalence of approximately 29% (95% CI 26%-32%) [5,6].

The early identification of DCI risk continues to pose significant challenges in clinical practice, with a notable absence of recognized and effective early predictive tools. Previous studies have explored diverse methods for the early prediction of DCI, such as transcranial Doppler (TCD) ultrasonography [7], computed tomography perfusion parameters [8], and continuous cranial electroencephalography monitoring [9]. Recent advancements in computational technologies and the ongoing improvement of statistical theory have facilitated the integration of artificial intelligence into clinical practice, mainly for the diagnosis of disease states, assessment of disease courses, and prediction of prognosis [10]. In this context, some studies have attempted to develop machine learning (ML) models for the early prediction of DCI after SAH; however, its accuracy remains debatable [11-14]. In addition, there is a lack of systematic investigations on effective predictors for the early prediction of DCI. Therefore, we conducted this meta-analysis to systematically discuss the accuracy of ML models in DCI prediction after SAH and to offer evidence for advancing the application of artificial intelligence in this field.

## Methods

### Study Registration

This study follows the PRISMA (Preferred Reporting Items for Systematic Reviews and Meta-Analyses) checklist [15]. This study protocol was registered in the International Prospective Register of Systematic Reviews on July 6, 2023 (CRD42023438399).

### Eligibility Criteria

The inclusion criteria were as follows: (1) patients with SAH, with the outcome event being DCI; (2) complete construction of an ML model for DCI prediction; (3) primary studies even without independent external validation or any kind of independent validation set; (4) published studies that constructed different ML models based on the same dataset; (5) cohort studies, case-control studies, or cross-sectional studies; and (6) studies published in English. The exclusion criteria were as follows: (1) meta-analysis, guidelines, review, or expert comments; (2) primary studies that analyzed risk factors/independent predictors only, without complete construction of an ML model; (3) absence of any outcome indicators for estimating the prediction accuracy of ML models, including the area under the receiver operating characteristic curve, concordance index (C index), accuracy, sensitivity, specificity, precision, confusion matrix, and *F*_1_-score; and (4) studies with sample size <20.

### Data Sources and Search Strategy

PubMed, Cochrane, Embase, and Web of Science databases were systematically searched up to May 18, 2023, by using Medical Subject Headings (MeSH) and free-text words, with no restrictions on publication year or country. The search strategy is described in Multimedia Appendix 1.

### Study Selection and Data Retrieval

The retrieved studies were imported into EndNote, and duplicates were excluded through software automarking and manual marking. Then, the titles or abstracts of the remaining studies were scanned, followed by full-text reviews to obtain the final eligible studies. A standardized table was established before data retrieval, and the extracted information included title, first author, publication year and country, study type, DCI cases, total cases, DCI cases in the training and validation sets, cases in the training and validation sets, generation method of the validation set, methods for tackling overfitting and missing data, variable screening methods, model type, and predictors. Literature screening was implemented independently by 2 researchers (HZ and PZ) and then cross-checked. Debatable studies were discussed with a third researcher (PL) to make a final decision.

### Assessment of Study Quality

The risk of bias in the primary studies was appraised using PROBAST (Prediction Model Risk of Bias Assessment Tool), which contains many questions on participants, predictors, outcomes, and statistical analyses, reflecting the overall risk of bias and applicability [16]. These domains had 2, 3, 6, and 9 specific questions, with 3 responses (yes/probably yes, no/probably no, and no information) for each question. A domain was regarded as having a high risk of bias if it had at least one no/probably no answer or a low risk of bias if all answers were yes or probably yes. The overall risk was assessed as low with all domains at low risk and high with at least one domain at high risk. Two researchers (HZ and PZ) assessed the risk of bias independently based on PROBAST and cross-checked at the end. If there was a debatable result, a third researcher (PL) was asked to assist in the assessment.

### Outcome Indicators

Currently, the C index is an important outcome index for assessing the prediction accuracy of ML models. However, in case of severe imbalance in case number (grossly too few cases for DCI), the C index can hardly explain the specific accuracy of the model for both positive and negative events, and thus, our primary outcome indicators also included sensitivity and specificity at the optimal thresholds of the model.

### Statistical Analysis

In some primary studies, when 95% CIs and standard errors were absent in the C index, we referred to Debray et al [17] to assess its standard error. As ML models contained different variables and inconsistent parameters, we gave priority to random-effects models in the meta-analysis of C index. The C index is currently an important end point metric for evaluating ML performance, especially in binary classification and survival analysis [18,19]. Many current binary classification ML studies have used the C index as the main metric for assessing model performance. In addition, ML obtains the probability of positive events. We also need to understand the sensitivity and specificity of ML prediction of positive events under the optimal risk probability cutoff threshold. Therefore, we need to perform a meta-analysis of the sensitivity and specificity based on the diagnostic 2×2 table.

A bivariate mixed-effects model was applied for the meta-analysis of sensitivity and specificity based on the diagnostic fourfold table. Most primary studies did not report the diagnostic fourfold table; therefore, the following 2 ways were used to calculate the diagnostic fourfold table: (1) using sensitivity, specificity, precision, and case number and (2) using sensitivity and specificity extracted based on the optimal Youden index as well as case number. In the meta-analysis of the C index, sensitivity and specificity evaluation were performed in Stata 15.0, and the program packages involved were metan and midas. The meta-analysis of the incidence of DCI was performed in R4.4.1 (R development Core Team). Because of the meta-analysis of the incidence, we need to transform the distribution of the incidence, and the R language packages involved are metafor and meta.

## Results

### Study Selection

**Figure 1:**
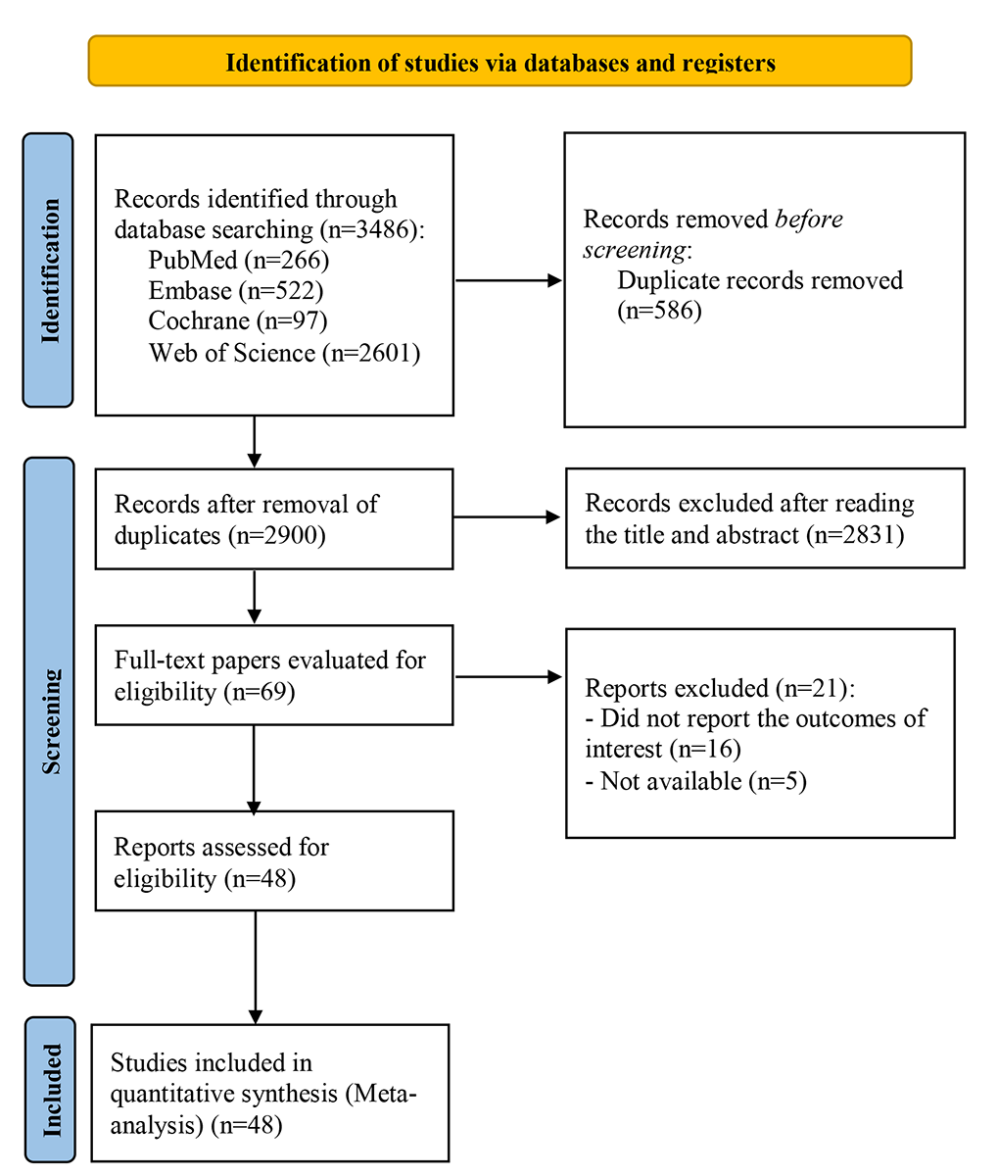
PRISMA (Preferred Reporting Items for Systematic Reviews and Meta-Analyses) literature screening flowchart.

We searched 3486 studies (97 from Cochrane, 266 from PubMed, 522 from Embase, and 2601 from Web of Science) and eliminated 586 duplicates. After reading the title or abstract, we excluded 2831 studies. Among the remaining 69 studies, 48 eligible primary studies [11-14,20-63] were finally included after reviewing the full texts (Figure 1).

### Study Characteristics

The 48 primary studies were published between 2013 and 2023, including 15,346 patients with SAH, of which 3863 patients had DCI. These primary studies were from 13 countries, including the United States [11-13, 21,24, 28, 31, 33, 36, 37, 46, 47, 49-51, 55, 57], China [20, 22, 23, 25, 26, 30, 35, 39, 41, 44, 53, 59], and the Netherlands [40,48,58,62]. In addition to one case-control study [11], the others were cohort studies. Ten studies were multicentered [24-26,28,37,39,40,42,58,61], and the remaining were one-centered. Besides, 21 studies [14, 21, 22, 27, 28, 30, 32, 33, 43, 45-47, 50, 52, 56-61, 63] lacked an independent validation set, and 25 studies [12, 20, 21, 23, 25, 26, 31, 35-42, 45, 47-51, 53-55, 62] reported the methods to avoid overfitting. Nineteen studies [12, 14, 20, 22, 24-26, 30, 31, 33, 34, 36-38, 48-51, 62] had missing data, which were mainly resolved by deletion, mean value imputation, and missing value imputation. In terms of variable screening, only 1 study [50] used a single-factor screening method (Multimedia Appendix 2).

### Risk of Bias

**Figure 2:**
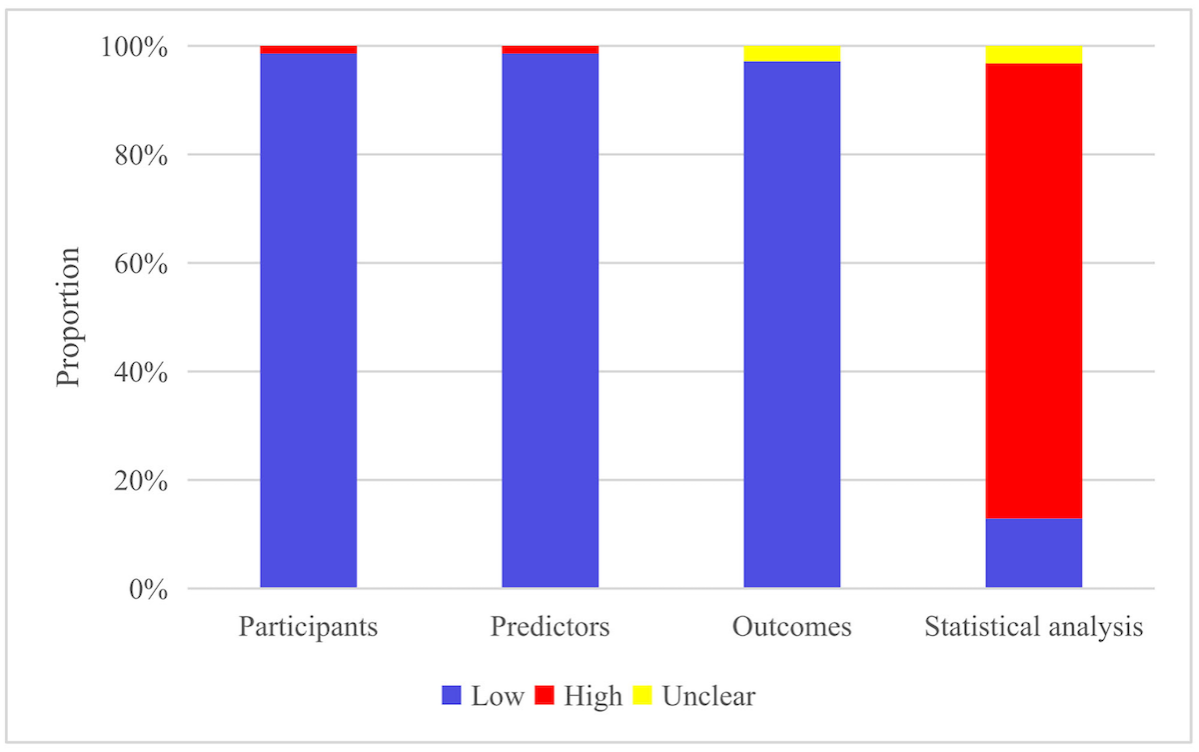
Risk of bias of the included machine learning models.

The 48 primary studies contained 71 ML models. In the assessment of participants and predictors, a high risk of bias was mainly derived from the ML models constructed in 1 case-control study. In the assessment of outcomes, only 1 model in the case-control study was rated as unclear risk. As for the statistical analyses, the models were mainly rated at high risk since most models did not satisfy modeling sample size estimation, that is, events per variable were >20 in the training set (DCI cases were greater than 20 times that were ultimately included in the model) and needed an independent validation set with over 100 validated cases. In addition, the methods for tackling overfitting and underfitting were not clearly described. The methods for handling missing data during quality assessment remained a concern, as PROBAST only recognized the multiple imputation method as low risk of bias (Figure 2).

### Meta-Analysis

**Figure 3:**
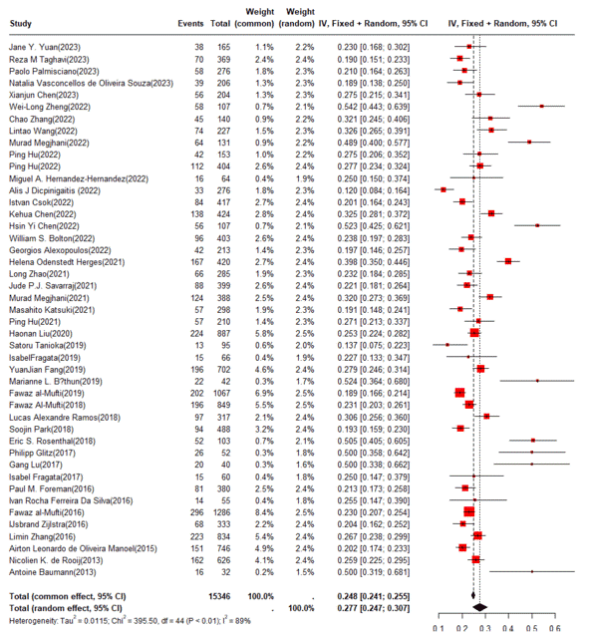
Forest plot of delayed cerebral ischemia incidence in the training set. IV: independent variable.

Of the 48 studies included, 45 explicitly reported the numbers of DCI and total SAH, and the prevalence of DCI, which was pooled using a random-effects model, was 27.7% (95% CI 24.7%-30.7%) (Figure 3 [11-14,20-39,41-50,52-59,61-63]).

### Newly Developed ML Models

#### C Index

**Figure 4:**
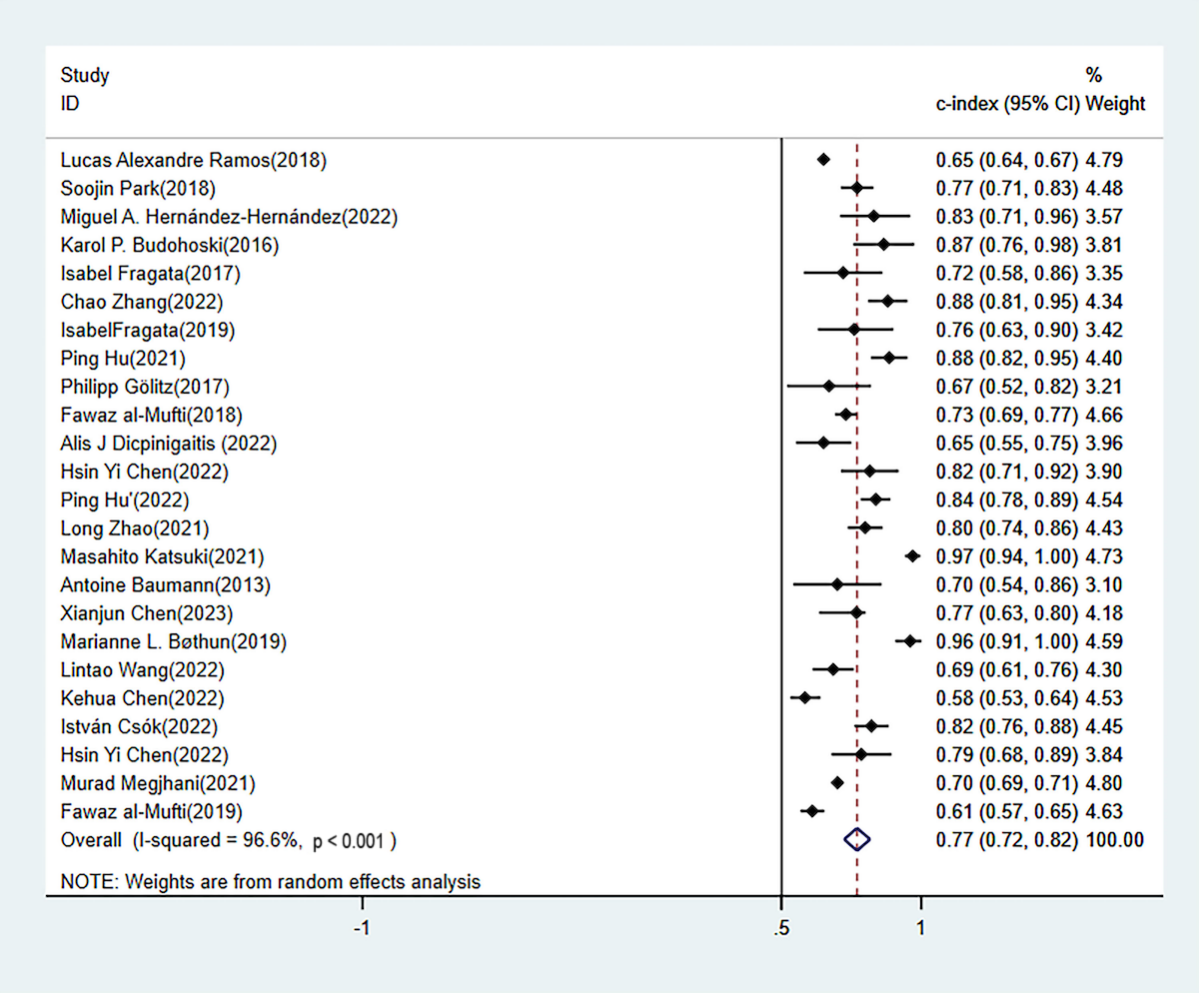
Forest plot of the concordance index of other machine learning models in the training set.

**Figure 5:**
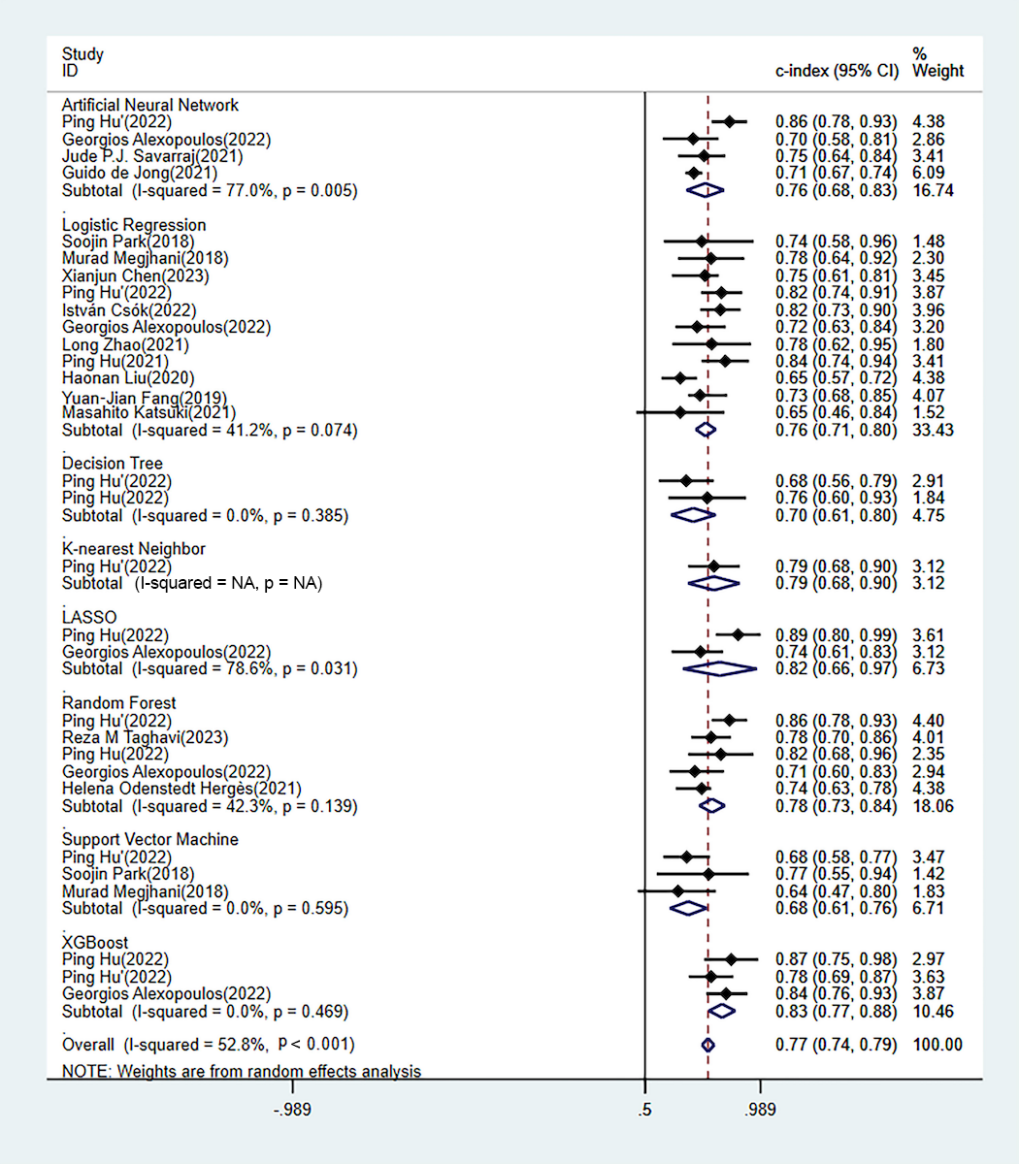
Forest plot of the concordance index of the machine learning models in the validation set. LASSO: least absolute shrinkage and selection operator; XGBoost: extreme gradient boosting.

In the training set, C index was reported in 45 ML models, with an overall C index of 0.786 (95% CI 0.737-0.835). The 31 independent validation cohorts manifested an overall C index of 0.767 (95% CI 0.741-0.793). Among them, the logistic regression–based predictive nomogram was the main model, with an overall C index of 0.770 (95% CI 0.724-0.817) in the training set and 0.757 (95% CI 0.715-0.800) in the validation set (Table 1, Figure 4 [1,26,27,32,37,38,41,42,44,49,50,54], Figure 5 [12,20,26,27,30,34-37,39-43,50,52]).

**Table 1:** Meta-analysis of the concordance index of machine learning models for delayed cerebral ischemia prediction in the training and validation sets.

Machine learning model	Training set		Validation set	
	n	Concordance index (95% CI)	n	Concordance index (95% CI)
Artificial neural network	4	0.721 (0.660-0.783)	4	0.756 (0.676-0.835)
Logistic regression	24	0.770 (0.724-0.817)	11	0.757 (0.715-0.800)
Decision tree	2	0.829 (0.782-0.877)	2	0.705 (0.610-0.799)
Ensemble classifier	1	0.830 (0.795-0.865)	—^a^	—
K-nearest neighbor	1	0.992 (0.986-0.999)	1	0.792 (0.683-0.901)
LASSO (least absolute shrinkage and selection operator)	1	0.793 (0.692-0.893)	2	0.817 (0.661-0.973)
Random forest	5	0.770 (0.646-0.894)	5	0.784 (0.730-0.838)
Support vector machine	5	0.789 (0.619-0.959)	3	0.684 (0.606-0.761)
Extreme gradient boosting	2	0.906 (0.860-0.952)	3	0.826 (0.770-0.882)
Overall	45	0.786 (0.737-0.835)	31	0.767 (0.741-0.793)

^a^Not available.

#### Sensitivity and Specificity

**Figure 6:**
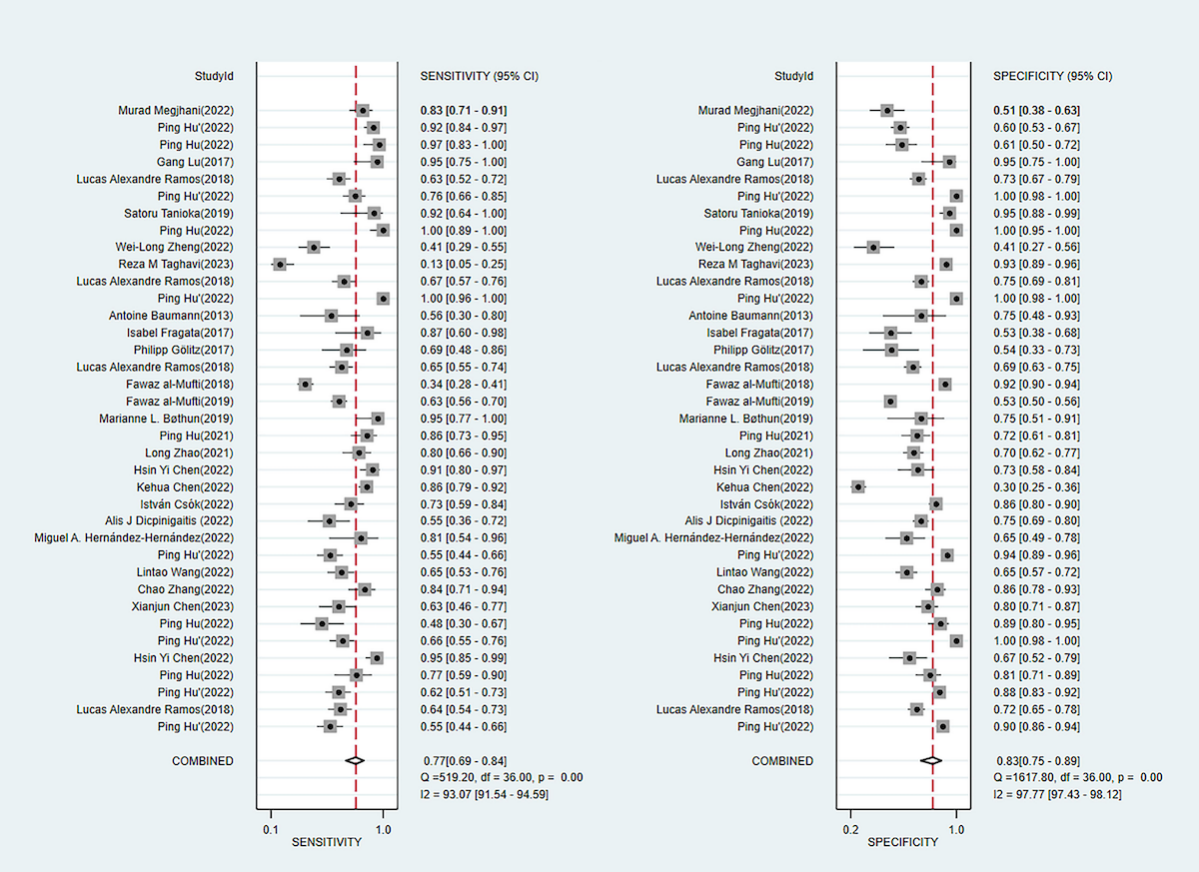
Forest plot of the sensitivity and specificity of the machine learning models in the training set.

**Figure 7:**
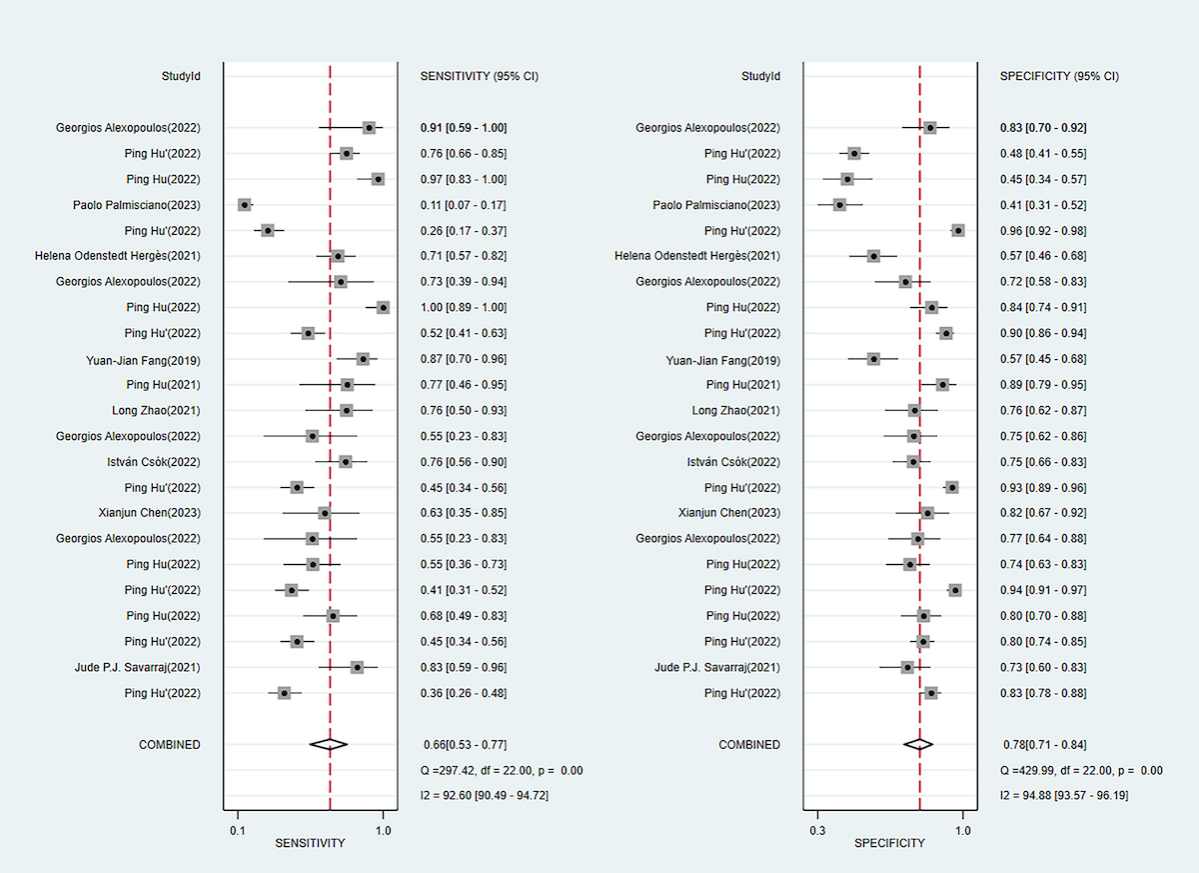
Forest plot of the sensitivity and specificity of the machine learning models in the validation set.

In the training set, we extracted diagnostic 2×2 tables from 37 ML models, with overall sensitivity of 0.77 (95% CI 0.69-0.84) and overall specificity of 0.83 (95% CI 0.75-0.89). The diagnostic 2×2 tables were also extracted from 23 independent validation cohorts, with overall sensitivity of 0.66 (95% CI 0.53-0.77) and overall specificity of 0.78 (95% CI 0.71-0.84). Among them, the logistic regression–based predictive nomogram had overall sensitivity of 0.75 (95% CI 0.67-0.82) and overall specificity of 0.71 (95% CI 0.63-0.78) in the training set and 0.59 (95% CI 0.57-0.80) and 0.80 (95% CI 0.71-0.87), respectively, in the validation set (Table 2, Figure 6 [12,20-22,24-32,36,40,43,46-49,53-55,63], Figure 7 [13,20,26,27,30,34-37,40,45]).

**Table 2:** Meta-analysis of the sensitivity and specificity of the machine learning models for delayed cerebral ischemia prediction in the training and validation sets.

Machine learning model	Training set				Validation set		
	n	Sensitivity (95% CI)	Specificity (95% CI)	n		Sensitivity (95% CI)	Specificity (95% CI)
Artificial neural network	2	0.55-0.64	0.72-0.91	2		0.37-0.82	0.72-0.84
Logistic regression	20	0.75 (0.67-0.82)	0.71 (0.63-0.78)	5		0.59 (0.57-0.80)	0.80 (0.71-0.87)
Decision tree	2	0.62-0.78	0.82-0.88	2		0.44-0.67	0.80-0.81
K-nearest neighbor	1	0.66	1	1		0.41	0.95
LASSO (least absolute shrinkage and selection operator)	1	0.48	0.89	2		0.54-0.55	0.74-0.78
Random forest	6	0.93 (0.28-1.00)	0.97 (0.66-1.00)	4		0.81 (0.46-0.96)	0.78 (0.63-0.88)
Support vector machine	3	0.63-0.95	0.73-1.00	1		0.26	0.96
Extreme gradient boosting	2	0.93-0.98	0.60-0.61	4		0.78 (0.27-0.97)	0.55 (0.37-0.71)
Overall	37	0.77 (0.69-0.84)	0.83 (0.75-0.89)	23		0.66 (0.53-0.77)	0.78 (0.71-0.84)

### Validation of Previous Scales

**Figure 8:**
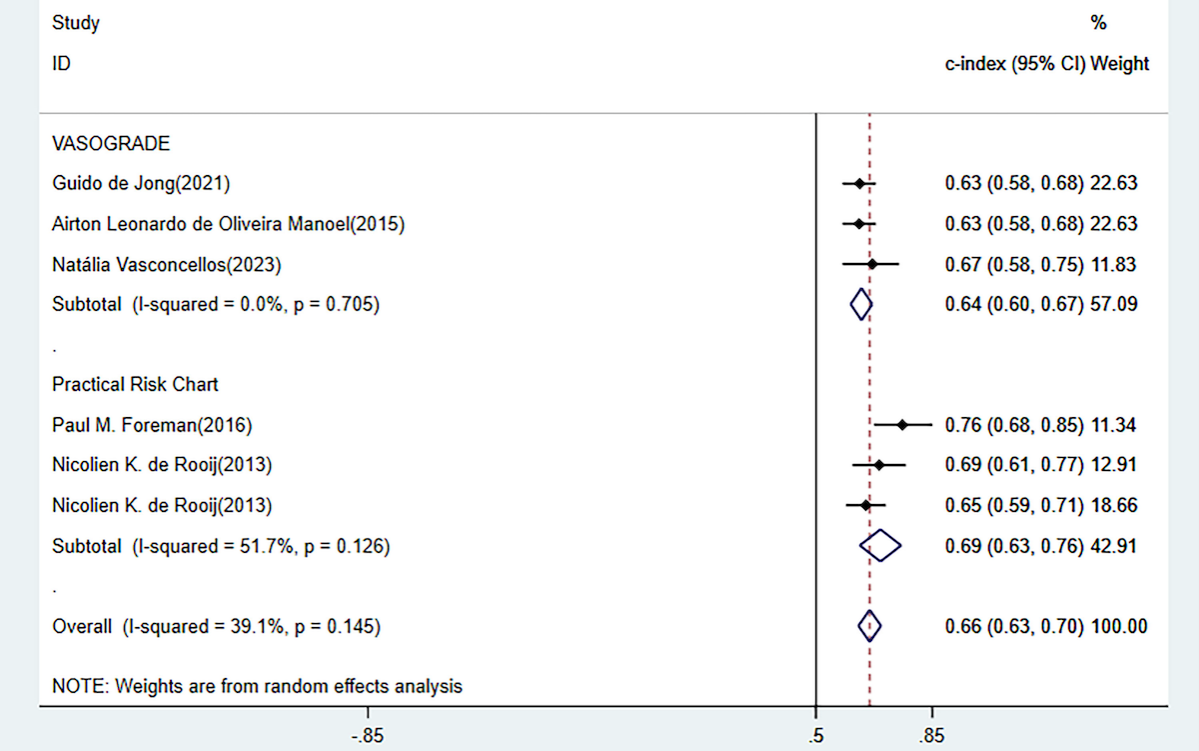
Forest plot of the concordance index of previous scales in the training set.

**Figure 9:**
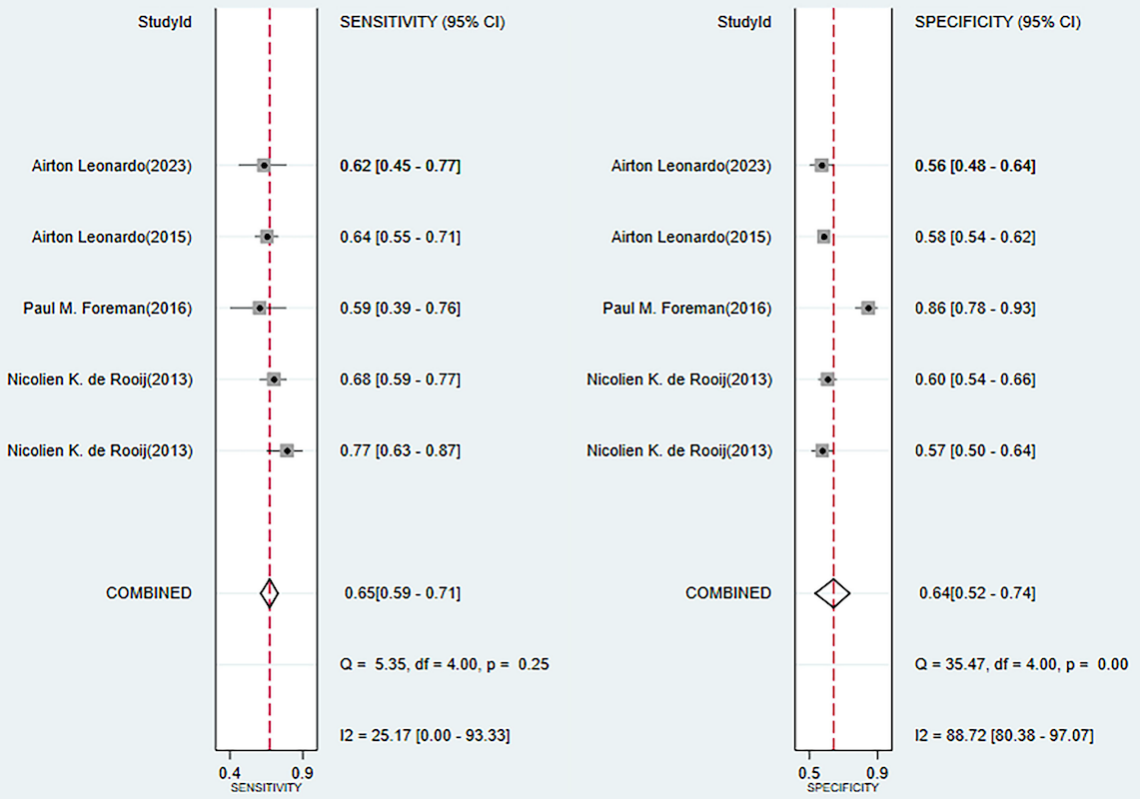
Forest plot of the sensitivity and specificity of previous scales in the training and validation sets.

The previous models mainly contained VASOGRADE and Practical Risk Chart, with an overall C index of 0.661 (95% CI 0.627-0.696), sensitivity of 0.65 (95% CI 0.59-0.87), and specificity of 0.64 (95% CI 0.52-0.74) (Figure 8 [14,41,56,62,63], Figure 9 [14,56,62,63]).

### Predictors

A total of 59 predictors were reported in the included ML models, and the top 10 were age (53 models), gender (46 models), Hunt and Hess score (41 models), modified Fisher scale (40 models), World Federation of Neurological Surgeons (35 models), aneurysm location (32 models), hypertension (29 models), coiling or clipping (28 models), smoking/drinking history (28 models), diabetes (23 models), and laboratory results (21 models). All modeling predictors are shown in Multimedia Appendix 3.

## Discussion

### Main Findings

Our study systematically explores the accuracy of ML for DCI prediction after SAH, and the results showed that ML had a relatively ideal C index for DCI prediction; however, the sensitivity still needs improving. The logistic regression–based nomogram was the main model. The predictors in the 71 models were mainly age, gender, Hunt and Hess score, modified Fisher scale, World Federation of Neurological Surgeons, aneurysm location, hypertension, coiling or clipping, smoking/drinking history, diabetes, and laboratory results.

### Comparison With Previous Studies

Continuous investigations have been undertaken to find reliable approaches for the early detection of DCI risk, mainly focusing on imaging techniques. Kumar et al [7] conducted a systematic review of 17 primary studies and reported the accuracy of TCD for predicting vasospasm for the diagnosis of DCI, with an overall 90% (95% CI 77%-96%) sensitivity and 71% (95% CI 51%-84%) specificity. Although their study demonstrated high sensitivity and relatively good specificity, we found that they included many retrospective studies, which may exaggerate the results. Therefore, more randomized diagnostic trials are also needed for validation. Han et al [8] provided evidence for the early prediction of DCI by using computed tomography perfusion parameters, with 97% sensitivity and 89% specificity. A systematic review by Scherschinski et al [9] described the prediction accuracy of continuous cranial electroencephalography monitoring for DCI (sensitivity 11.1%, 95% CI 3.1%-26.1% and specificity 82.9%, 95% CI 66.4%-93.4%). In addition, a systematic review by Kumar et al [64] described the prediction of DCI by cerebral angiography (sensitivity 57%, 95% CI 40%-81%; specificity 68%, 95% CI 61%-76%). Of these imaging tools, Doppler ultrasound appears to have high prediction accuracy, especially with high sensitivity.

Furthermore, Santana et al [65] reported the accuracy of ML in DCI prediction after SAH through a systematic review, which, however, only included 6 primary studies. Their results demonstrated that the overall sensitivity of logistic regression was 0.71 (95% CI 0.57-0.84) and specificity was 0.63 (95% CI 0.42-0.85), and other ML models had a pooled sensitivity of 0.74 (95% CI 0.61-0.86) and specificity of 0.78 (95% CI 0.71-0.86). However, the training and validation set results did not seem to be considered in their studies. In our systematic review incorporating 48 primary studies, the overall C index in the validation set was 0.767 (95% CI 0.741-0.793), with sensitivity of 0.66 (95% CI 0.53-0.77) and specificity of 0.78 (95% CI 0.71-0.84). Meanwhile, logistic regression was the main model, with low sensitivity in the validation set.

In clinical practice, the interpretability of the model and the prediction accuracy for outcomes should be considered; however, it is hard to choose between them [66]. Some models with high interpretability, that is, logistic regression (converting linear combinations of predictor variables into probability values for positive events via logistic functions), decision trees (generating rules by recursively splitting variables in the dataset), and LASSO (least absolute shrinkage and selection operator) regression (selecting variables and reducing model complexity by L1 regularization), which adds a penalty term proportional to the absolute value of coefficients of the features for feature selection and model construction, often have too low prediction accuracy for outcomes. Others with too little explainability, that is, extreme gradient boosting (XGBoost; combining multiple weak learners to form a strong learner with efficient computational power and excellent prediction performance), artificial neural network (an algorithm that simulates the neuronal structure of the human brain and learns complex patterns of data through multilayer nonlinear transformations for various ML tasks such as classification, regression, and clustering), and support vector machines (a supervised learning method to maximize the spacing between different classes by finding optimal hyperplanes for classification and regression tasks) have very high prediction accuracy, especially deep learning models [67]. Among ML models constructed based on common clinical features, models with better interpretability are preferred, which can explicitly explain the relationship between common clinical features and outcomes and thus provide real-time interventions. This may also explain why logistic regression was most frequently used in the included studies. However, in image-based artificial intelligence, models with higher prediction efficiency are preferred, mainly since medical images have many confounding factors and types of imaging devices, as well as a high level of device parameterization [10,68]. Our study shows that XGBoost in the validation set had the best C index of 0.826 (95% CI 0.770-0.882), with sensitivity and specificity of 0.78 (95% CI 0.27-0.97) and 0.55 (95% CI 0.37-0.71), respectively. Although XGBoost can rank the variables’ importance, its interpretability remains a serious challenge. In addition, random forests seem to exhibit desirable accuracy, with C index, sensitivity, and specificity of 0.784 (95% CI 0.730-0.838), 0.81 (95% CI 0.46-0.96), and 0.78 (95% CI 0.63-0.88), respectively. The c-indices of logistic regression, LASSO regression, and decision tree based on models with better interpretability were 0.757 (0.715-0.800), 0.705 (0.610-0.799), and 0.817 (0.661-0.973), respectively. This shows the differences in the prediction accuracy of different models, where the less interpretable XGBoost and random forest seem to have better accuracy, especially random forest, which has desirable sensitivity and specificity.

Our study shows that existing ML has a more favorable C index for the prediction of DCI; however, the overall sensitivity seems to be unfavorable. Although random forest has high sensitivity and specificity, the included models are limited, which may limit the interpretation of results. In constructing ML models, we need to consider data balance. For unbalanced modeling, some results tend to show a high C index, which is due to negative results. Our pooled incidence of DCI for the included studies was 27.7% (95% CI 24.7%-30.7%), which may have some implications for low sensitivity. In addition, some models obtain the probability of DCI, and the probability thresholds are often chosen based on the optimal Youden index. The effect of sensitivity is often not considered when choosing the optimal Youden index, which may also have some effect on low sensitivity. Therefore, the balance of modeling data and the reasonableness of positive probability thresholds need to be considered in future studies.

When constructing clinical prediction models, the number of included cases and the selection of predictors are often neglected. Models derived from a restricted number of cases exhibited low stability, and some included studies had a limited sample size. In addition, efficient predictors are key to enhancing model accuracy, and our summary of the current modeling predictors provides important references for subsequent studies. A systematic review by Jabbarli et al [69] explored the biomarkers for the early prediction of DCI, which greatly differed from the markers we summarized. Efficient predictors should be further explored in subsequent studies.

### Limitations of This Study

Our study has the following limitations. First, there may be some differences in the definition of DCI among studies, which may impose some limitations on the interpretation of our results to some extent. Second, the included studies contain a diversity of models. However, except for logistic regression, the other types of models are limited in number. Third, there is a high risk of bias in primary studies, mainly owing to the limited cases for modeling and the avoidance of overfitting. Fourth, the validation methods for the models in the primary studies are mainly random sampling or k-fold cross-validation, with rare external validation. Fifth, in a very small number of studies, validation sets can be used to try out different hyperparameters to improve the algorithm; test sets are essentially raw data. It is not clear in the original studies we included whether the models were optimized in this way. Therefore, we were not able to discuss this in greater depth in our review. Sixth, even though we conducted a systematic search and literature screening, no studies were found on image-based ML for predicting DCI in patients with SAH. Therefore, deeper exploration should be conducted in future studies.

### Conclusion

The ML constructed in the original studies that we included was primarily based on the baseline characteristics of patients at admission. Our meta-analysis shows that ML had a relatively desirable predictive value. In subsequent studies, more efficient predictors can be explored and better-performing prediction tools can be developed based on the ML approach to enable treating physicians to enhance daily TCD monitoring. Prioritizing TCD monitoring in resource-limited settings lowers the threshold for performing more invasive diagnostic procedures such as catheter angiography.

## Appendix

Multimedia Appendix 1 Literature search strategy.

Multimedia Appendix 2 Basic study characteristics.

Multimedia Appendix 3 Modeling predictors.

Multimedia Appendix 4 PRISMA (Preferred Reporting Items for Systematic reviews and Meta-Analyses) 2020 checklist.

## Abbreviations

DCI: delayed cerebral ischemia
LASSO: least absolute shrinkage and selection operator
MeSH: Medical Subject Headings
ML: machine learning
PRISMA: Preferred Reporting Items for Systematic Reviews and Meta-Analyses
PROBAST: Prediction Model Risk of Bias Assessment Tool
SAH: subarachnoid hemorrhage
TCD: transcranial Doppler
XGBoost: extreme gradient boosting

## Data Availability

The original contributions presented in this study are included in this paper and the multimedia appendices. Further inquiries can be directed to the corresponding author.
