# Supplementary material for: Machine Learning for the Early Prediction of Delayed Cerebral Ischemia in Patients With Subarachnoid Hemorrhage: Systematic Review and Meta-Analysis
Source: J Med Internet Res. 2025 Jan 20;27:e54121. doi: 10.2196/54121 (PMC11791451; doi:10.2196/54121)
Supplement: Multimedia Appendix 1 [file jmir_v27i1e54121_app1.docx]

# Multimedia Appendix 1 Literature search strategy

**1.Pubmed**

| Search number | Query | Results |
| --- | --- | --- |
| #1 | ((((((((((((((((((((((((((((Subarachnoid Hemorrhage[MeSH Terms]) OR (Subarachnoid Hemorrhage[Title/Abstract])) OR (Subarachnoid Hemorrhages[Title/Abstract])) OR (Aneurysmal Subarachnoid Haemorrhage[Title/Abstract])) OR (Aneurysmal Subarachnoid Hemorrhage[Title/Abstract])) OR (Aneurysmal Subarachnoid Hemorrhages[Title/Abstract])) OR (Arachnoidal Bleeding[Title/Abstract])) OR (Arachnoidal Haemorrhage[Title/Abstract])) OR (Arachnoidal Hemorrhage[Title/Abstract])) OR (Brain Arachnoid Haemorrhage[Title/Abstract])) OR (Brain Arachnoid Hemorrhage[Title/Abstract])) OR (Intracranial Subarachnoid Hemorrhage[Title/Abstract])) OR (Intracranial Subarachnoid Hemorrhages[Title/Abstract])) OR (Perinatal Subarachnoid Hemorrhage[Title/Abstract])) OR (Perinatal Subarachnoid Hemorrhages[Title/Abstract])) OR (Subarachnoid Bleeding[Title/Abstract])) OR (Subarachnoid Blood[Title/Abstract])) OR (Subarachnoid Haematoma[Title/Abstract])) OR (Subarachnoid Haemorrhage[Title/Abstract])) OR (Subarachnoid Hematoma[Title/Abstract])) OR (Subarachnoid Hemorrhagia[Title/Abstract])) OR (Subarachnoidal Bleeding[Title/Abstract])) OR (Subarachnoidal Haemorrhage[Title/Abstract])) OR (Subarachnoidal Hemorrhage[Title/Abstract])) OR (Spontaneous Subarachnoid Haemorrhage[Title/Abstract])) OR (Spontaneous Subarachnoid Hemorrhage[Title/Abstract])) OR (Spontaneous Subarachnoid Hemorrhages[Title/Abstract])) OR (Traumatic Subarachnoid Haemorrhage[Title/Abstract])) OR (Traumatic Subarachnoid Hemorrhage[Title/Abstract]) | 36,070 |
| #2 | ((((((((((((((((((((((((((Machine Learning[MeSH Terms]) OR (Machine Learning[Title/Abstract])) OR (Transfer Learning[Title/Abstract])) OR (Deep Learning[Title/Abstract])) OR (Ensemble Learning[Title/Abstract])) OR (Artificial Intelligence[Title/Abstract])) OR (Random Forest[Title/Abstract])) OR (Neural Network[Title/Abstract])) OR (Neural Networks[Title/Abstract])) OR (K-Nearest Neighbor[Title/Abstract])) OR (CNN[Title/Abstract])) OR (Support Vector Machine[Title/Abstract])) OR (SVM[Title/Abstract])) OR (Gradient Boosting Machine[Title/Abstract])) OR (Nomogram[Title/Abstract])) OR (XGBoost[Title/Abstract])) OR (Adaboost[Title/Abstract])) OR (Decision Tree[Title/Abstract])) OR (ResNet-50[Title/Abstract])) OR (ResNet[Title/Abstract])) OR (Naive Bayesian[Title/Abstract])) OR (Multilayer Perceptron[Title/Abstract])) OR (Bayesian network[Title/Abstract])) OR (Radiomics[Title/Abstract])) OR (Radiomic[Title/Abstract])) OR (Prediction Model[Title/Abstract])) OR (Risk Model[Title/Abstract]) | 284,645 |
| #3 | (((((((((((((((((((((((((((((Subarachnoid Hemorrhage[MeSH Terms]) OR (Subarachnoid Hemorrhage[Title/Abstract])) OR (Subarachnoid Hemorrhages[Title/Abstract])) OR (Aneurysmal Subarachnoid Haemorrhage[Title/Abstract])) OR (Aneurysmal Subarachnoid Hemorrhage[Title/Abstract])) OR (Aneurysmal Subarachnoid Hemorrhages[Title/Abstract])) OR (Arachnoidal Bleeding[Title/Abstract])) OR (Arachnoidal Haemorrhage[Title/Abstract])) OR (Arachnoidal Hemorrhage[Title/Abstract])) OR (Brain Arachnoid Haemorrhage[Title/Abstract])) OR (Brain Arachnoid Hemorrhage[Title/Abstract])) OR (Intracranial Subarachnoid Hemorrhage[Title/Abstract])) OR (Intracranial Subarachnoid Hemorrhages[Title/Abstract])) OR (Perinatal Subarachnoid Hemorrhage[Title/Abstract])) OR (Perinatal Subarachnoid Hemorrhages[Title/Abstract])) OR (Subarachnoid Bleeding[Title/Abstract])) OR (Subarachnoid Blood[Title/Abstract])) OR (Subarachnoid Haematoma[Title/Abstract])) OR (Subarachnoid Haemorrhage[Title/Abstract])) OR (Subarachnoid Hematoma[Title/Abstract])) OR (Subarachnoid Hemorrhagia[Title/Abstract])) OR (Subarachnoidal Bleeding[Title/Abstract])) OR (Subarachnoidal Haemorrhage[Title/Abstract])) OR (Subarachnoidal Hemorrhage[Title/Abstract])) OR (Spontaneous Subarachnoid Haemorrhage[Title/Abstract])) OR (Spontaneous Subarachnoid Hemorrhage[Title/Abstract])) OR (Spontaneous Subarachnoid Hemorrhages[Title/Abstract])) OR (Traumatic Subarachnoid Haemorrhage[Title/Abstract])) OR (Traumatic Subarachnoid Hemorrhage[Title/Abstract])) AND (((((((((((((((((((((((((((Machine Learning[MeSH Terms]) OR (Machine Learning[Title/Abstract])) OR (Transfer Learning[Title/Abstract])) OR (Deep Learning[Title/Abstract])) OR (Ensemble Learning[Title/Abstract])) OR (Artificial Intelligence[Title/Abstract])) OR (Random Forest[Title/Abstract])) OR (Neural Network[Title/Abstract])) OR (Neural Networks[Title/Abstract])) OR (K-Nearest Neighbor[Title/Abstract])) OR (CNN[Title/Abstract])) OR (Support Vector Machine[Title/Abstract])) OR (SVM[Title/Abstract])) OR (Gradient Boosting Machine[Title/Abstract])) OR (Nomogram[Title/Abstract])) OR (XGBoost[Title/Abstract])) OR (Adaboost[Title/Abstract])) OR (Decision Tree[Title/Abstract])) OR (ResNet-50[Title/Abstract])) OR (ResNet[Title/Abstract])) OR (Naive Bayesian[Title/Abstract])) OR (Multilayer Perceptron[Title/Abstract])) OR (Bayesian network[Title/Abstract])) OR (Radiomics[Title/Abstract])) OR (Radiomic[Title/Abstract])) OR (Prediction Model[Title/Abstract])) OR (Risk Model[Title/Abstract])) | 266 |

**2.Cochrane**

| Search number | Query | Results |
| --- | --- | --- |
| #1 | MeSH descriptor: [Subarachnoid Hemorrhage] explode all trees | 826 |
| #2 | (Subarachnoid Hemorrhage):ti,ab,kw OR (Subarachnoid Hemorrhages):ti,ab,kw OR (Aneurysmal Subarachnoid Haemorrhage):ti,ab,kw OR (Aneurysmal Subarachnoid Hemorrhage):ti,ab,kw OR (Aneurysmal Subarachnoid Hemorrhages):ti,ab,kw (Word variations have been searched) | 2302 |
| #3 | (Arachnoidal Bleeding):ti,ab,kw OR (Arachnoidal Haemorrhage):ti,ab,kw OR (Arachnoidal Hemorrhage):ti,ab,kw OR (Brain Arachnoid Haemorrhage):ti,ab,kw OR (Brain Arachnoid Hemorrhage):ti,ab,kw (Word variations have been searched) | 47 |
| #4 | (Intracranial Subarachnoid Hemorrhage):ti,ab,kw OR (Intracranial Subarachnoid Hemorrhages):ti,ab,kw OR (Perinatal Subarachnoid Hemorrhage):ti,ab,kw OR (Perinatal Subarachnoid Hemorrhages):ti,ab,kw OR (Subarachnoid Bleeding):ti,ab,kw (Word variations have been searched) | 937 |
| #5 | (Subarachnoid Blood):ti,ab,kw OR (Subarachnoid Haematoma):ti,ab,kw OR (Subarachnoid Haemorrhage):ti,ab,kw OR (Subarachnoid Hematoma):ti,ab,kw OR (Subarachnoid Hemorrhagia):ti,ab,kw (Word variations have been searched) | 2,857 |
| #6 | (Subarachnoidal Bleeding):ti,ab,kw OR (Subarachnoidal Haemorrhage):ti,ab,kw OR (Subarachnoidal Hemorrhage):ti,ab,kw OR (Spontaneous subarachnoid Haemorrhage):ti,ab,kw OR (Spontaneous Subarachnoid Hemorrhage):ti,ab,kw (Word variations have been searched) | 2,318 |
| #7 | (Spontaneous Subarachnoid Hemorrhages):ti,ab,kw OR (Traumatic Subarachnoid Haemorrhage):ti,ab,kw OR (Traumatic Subarachnoid Hemorrhage):ti,ab,kw (Word variations have been searched) | 304 |
| #8 | #1 OR #2 OR #3 OR #4 OR #5 OR #6 OR #7 | 2881 |
| #9 | MeSH descriptor: [Machine Learning] explode all trees | 880 |
| #10 | (Machine Learning):ti,ab,kw OR (Transfer Learning):ti,ab,kw OR (Deep Learning):ti,ab,kw OR (Ensemble Learning):ti,ab,kw OR (Artificial Intelligence):ti,ab,kw (Word variations have been searched) | 6,818 |
| #11 | (Random Forest):ti,ab,kw OR (Neural Network):ti,ab,kw OR (Neural Networks):ti,ab,kw OR (K-Nearest Neighbor):ti,ab,kw OR (CNN):ti,ab,kw (Word variations have been searched) | 5,369 |
| #12 | (Support Vector Machine):ti,ab,kw OR (SVM):ti,ab,kw OR (Gradient Boosting Machine):ti,ab,kw OR (Nomogram):ti,ab,kw OR (XGBoost):ti,ab,kw (Word variations have been searched) | 2,205 |
| #13 | (Adaboost):ti,ab,kw OR (Decision Tree):ti,ab,kw OR (ResNet-50):ti,ab,kw OR (ResNet):ti,ab,kw OR (Naive Bayesian):ti,ab,kw (Word variations have been searched) | 1,237 |
| #14 | (Multilayer Perceptron):ti,ab,kw OR (Bayesian Network):ti,ab,kw OR (Radiomics):ti,ab,kw OR (Radiomic):ti,ab,kw OR (Prediction Model):ti,ab,kw (Word variations have been searched) | 27,239 |
| #15 | (Risk Model):ti,ab,kw (Word variations have been searched) | 48,197 |
| #16 | #8 OR #9 OR #10 OR #11 OR #12 OR #13 OR #14 | 74,692 |
| #17 | #15 AND #16 | 97 |

**3.Embase**

| Search number | Query | Results |
| --- | --- | --- |
| #1 | 'subarachnoid hemorrhage'/exp | 53,371 |
| #2 | 'subarachnoid hemorrhage':ab,ti OR 'subarachnoid hemorrhages':ab,ti OR 'aneurysmal subarachnoid haemorrhage':ab,ti OR 'aneurysmal subarachnoid hemorrhage':ab,ti OR 'aneurysmal subarachnoid hemorrhages':ab,ti OR 'arachnoidal bleeding':ab,ti OR 'arachnoidal haemorrhage':ab,ti OR 'arachnoidal hemorrhage':ab,ti OR 'brain arachnoid haemorrhage':ab,ti OR 'brain arachnoid hemorrhage':ab,ti OR 'intracranial subarachnoid hemorrhage':ab,ti OR 'intracranial subarachnoid hemorrhages':ab,ti OR 'perinatal subarachnoid hemorrhage':ab,ti OR 'perinatal subarachnoid hemorrhages':ab,ti OR 'subarachnoid bleeding':ab,ti OR 'subarachnoid blood':ab,ti OR 'subarachnoid haematoma':ab,ti OR 'subarachnoid haemorrhage':ab,ti OR 'subarachnoid hematoma':ab,ti OR 'subarachnoid hemorrhagia':ab,ti OR 'subarachnoidal bleeding':ab,ti OR 'subarachnoidal haemorrhage':ab,ti OR 'subarachnoidal hemorrhage':ab,ti OR 'spontaneous subarachnoid haemorrhage':ab,ti OR 'spontaneous subarachnoid hemorrhage':ab,ti OR 'spontaneous subarachnoid hemorrhages':ab,ti OR 'traumatic subarachnoid haemorrhage':ab,ti OR 'traumatic subarachnoid hemorrhage':ab,ti | 39,455 |
| #3 | #1 OR #2 | 58,271 |
| #4 | 'machine learning'/exp | 386,789 |
| #5 | 'machine learning':ab,ti OR 'transfer learning':ab,ti OR 'deep learning':ab,ti OR 'ensemble learning':ab,ti OR 'artificial intelligence':ab,ti OR 'random forest':ab,ti OR 'neural network':ab,ti OR 'neural networks':ab,ti OR 'k-nearest neighbor':ab,ti OR cnn:ab,ti OR 'support vector machine':ab,ti OR svm:ab,ti OR 'gradient boosting machine':ab,ti OR nomogram:ab,ti OR xgboost:ab,ti OR adaboost:ab,ti OR 'decision tree':ab,ti OR 'resnet 50':ab,ti OR resnet:ab,ti OR 'naive bayesian':ab,ti OR 'multilayer perceptron':ab,ti OR 'bayesian network':ab,ti OR radiomics:ab,ti OR radiomic:ab,ti OR 'prediction model':ab,ti OR 'risk model':ab,ti | 326,714 |
| #6 | #3 OR #4 | 526916 |
| #7 | #5 AND #6 | 522 |

**4.Web of science**

| Search number | Query | Results |
| --- | --- | --- |
| #1 | TS=(Subarachnoid Hemorrhage) OR TS=(Subarachnoid Hemorrhages) OR TS=(Aneurysmal Subarachnoid Haemorrhage) OR TS=(Aneurysmal Subarachnoid Hemorrhage) OR TS=(Aneurysmal Subarachnoid Hemorrhages) OR TS=(Arachnoidal Bleeding) OR TS=(Arachnoidal Haemorrhage) OR TS=(Arachnoidal Hemorrhage) OR TS=(Brain Arachnoid Haemorrhage) OR TS=(Brain Arachnoid Hemorrhage) OR TS=(Intracranial Subarachnoid Hemorrhage) OR TS=(Intracranial Subarachnoid Hemorrhages) OR TS=(Perinatal Subarachnoid Hemorrhage) OR TS=(Perinatal Subarachnoid Hemorrhages) OR TS=(Subarachnoid Bleeding) OR TS=(Subarachnoid Blood) OR TS=(Subarachnoid Haematoma) OR TS=(Subarachnoid Haemorrhage) OR TS=(Subarachnoid Hematoma) OR TS=(Subarachnoid Hemorrhagia) OR TS=(Subarachnoidal Bleeding) OR TS=(Subarachnoidal Haemorrhage) OR TS=(Subarachnoidal Hemorrhage) OR TS=(Spontaneous Subarachnoid Haemorrhage) OR TS=(Spontaneous Subarachnoid Hemorrhage) OR TS=(Spontaneous Subarachnoid Hemorrhages) OR TS=(Traumatic Subarachnoid Haemorrhage) OR TS=(Traumatic Subarachnoid Hemorrhage) | 54,638 |
| #2 | TS=(Machine Learning) OR TS=(Transfer Learning) OR TS=(Deep Learning) OR TS=(Ensemble Learning) OR TS=(Artificial Intelligence) OR TS=(Random Forest) OR TS=(Neural Network) OR TS=(Neural Networks) OR TS=(K-Nearest Neighbor) OR TS=(CNN) OR TS=(Support Vector Machine) OR TS=(SVM) OR TS=(Gradient Boosting Machine) OR TS=(Nomogram) OR TS=(XGBoost) OR TS=(Adaboost) OR TS=(Decision Tree) OR TS=(ResNet-50) OR TS=(ResNet) OR TS=(Naive Bayesian) OR TS=(Multilayer Perceptron) OR TS=(Bayesian Network) OR TS=(Radiomics) OR TS=(Radiomic) OR TS=(Prediction Model) OR TS=(Risk Model) | 2,891,811 |
| #3 | #2 AND #1 | 2,601 |
