# Supplementary material for: Machine Learning for the Early Prediction of Delayed Cerebral Ischemia in Patients With Subarachnoid Hemorrhage: Systematic Review and Meta-Analysis
Source: J Med Internet Res. 2025 Jan 20;27:e54121. doi: 10.2196/54121 (PMC11791451; doi:10.2196/54121)
Supplement: Multimedia Appendix 2 [file jmir_v27i1e54121_app2.docx]

**Multimedia Appendix 2** Basic study characteristics

| No. | First author | Publication year | Author's country | Study type | Sources of patients | Definition of delayed cerebral ischemia | Case of delayed cerebral ischemia | Total case | Total cases in the training set | Generation way of the validation set | Total cases in the validation set | Method for handling missing data | Variable screening methods |
| --- | --- | --- | --- | --- | --- | --- | --- | --- | --- | --- | --- | --- | --- |
| 1 | Jane Y. Yuan [11] | 2023 | USA | case-control study | single-center study | (1) neurologic deterioration (new focal neurological deficits or decline in level of consciousness) not explained by other causes or occurring in the peri-procedural period and either confirmed by angiographic vasospasm or by response to hemodynamic therapies; and/or (2) delayed cerebral infarction on CT or magnetic resonance imaging—that is, hypodensity in a vascular distribution not present on postprocedural imaging. | 38 | 165 | 41 | internal verification | 4 |  | multi-factor test |
| 2 | Reza M Taghavi [12] | 2023 | USA | cohort study | single-center study | new neurologic deterioration that was not evident after aneurysm treatment and that could not be attributed to aneurysm re-bleeding, hydrocephalus, infection, seizure, hyponatremia, or other metabolic abnormality | 70 | 369 | 276 | external verification | 93 | deleted | multi-factor test |
| 3 | Paolo Palmisciano [13] | 2023 | USA | cohort study | single-center study | 1) new-onset neurologic deficits between days 4 and 12 after the aneurysm rupture, defined by worsening of the Glasgow Coma Scale score (GCS) by >= 2 points; and 2) CT or magnetic resonance imaging (MRI) demonstrating new ischemic infarcts since admission. | 58 | 276 |  | external verification | 276 |  | multi-factor test |
| 4 | Natália Vasconcellos de Oliveira Souza [14] | 2023 | Brazil | cohort study | single-center study | a new neurological focal sign or a worsening of the level of consciousness, presumably related to ischemia, that lasts for at least 1 h and that cannot be explained by other causes | 39 | 206 | 206 |  |  | deleted | multi-factor test |
| 5 | Xianjun Chen [20] | 2023 | China | cohort study | single-center study | a sequela caused by cerebral vasospasm alone, and some scholars have even defined DCI directly as a poor prognosis for the occurrence of cerebral vasospasm | 56 | 204 | 144 | internal verification | 60 | deleted | single-factor + multi-factor tests |
| 6 | Wei-Long Zheng [21] | 2022 | USA | cohort study | single-center study | (1) new focal neurologic deficits and/or decrease in the Glasgow Coma Scale of at least 2 points, !one hour, not explained by other causes (e.g. procedural complications, sedation, intracranial hypertension, hydrocephalus, systemic abnormalities) based on clinical assessment, imaging or laboratory data, or (2) the presence of cerebral infarction on CT or MRI imaging of the brain, that was not present on any neuroimaging done within the first 48 hours following early aneurysm occlusion, and not attributable to other causes such as surgical clipping or endovascular treatment. | 58 | 107 |  |  |  |  | multi-factor test |
| 7 | Chao Zhang [22] | 2022 | China | cohort study | single-center study | (1) occurrence of focal neurological impairment, or a decrease of at least 2 points on GCS and last at least 1 h, not apparent immediately after aneurysm occlusion, and not attributed to other conditions. (2) The presence of cerebral infarction on CT/MRI within 6 weeks, not present on imaging up to 48 hours after aneurysm occlusion, and not attributed to other causes such as surgical clipping or endovascular treatment. | 45 | 140 |  |  |  | deleted | single-factor + multi-factor tests |
| 8 | Lintao Wang [23] | 2022 | China | cohort study | single-center | DCI is a clinical imaging syndrome that includes focal ischemia and/or cognitive impairment on CT/MRI and/or cerebral infarction | 74 | 227 |  | internal verification |  |  | single-factor + multi-factor tests |
| 9 | Murad Megjhani [24] | 2022 | USA | cohort study | double-center | delayed neurological deterioration defined as a≥2-point change in GCS or new focal neurological deficit lasting for > 1 h and not associated with surgical treatment and/or a new cerebral infarct on brain imaging that is not attributable to any other causes defined a territorial or watershed deficit on CT perfusion unrelated to other causes as DCI | 64 | 131 |  | internal verification |  | median | multi-factor test |
| 10 | Ping Hu [25] | 2022 | China | cohort study | multi-center study | (1) No other cause could have led to the occurrence of a permanent or temporary focal neurological impairment (such as aphasia, apraxia, hemianopia, or neglect) between 4 and 14 days after aSAH; (2) the Glasgow Coma Scale score decreased by at least two points (either on one of its components [eye opening, verbal response, and motor response] or on total score); and (3) head CT scans revealed a low-density area that was not noticeable on admission or immediately after the operation, and there were no other causes except vasospasms between 4 and 30 days after aSAH | 42 | 153 | 111 | internal verification | 42 | deleted | multi-factor test |
| 11 | Ping Hu [26] | 2022 | China | cohort study | multi-center study | (1) no other etiology could have caused a permanent or temporary focal neurological impairment (such as aphasia, apraxia, hemianopia, or neglect) between 4 and 14 days after aSAH; (2) the Glasgow Coma Scale score decreased by at least two points [either on one of its components (eye opening, verbal response, motor response), or on total score]; and (3) head CT scans revealed a low-density area that was not noticeable on admission or immediately after the operation, and there were no other causes except vasospasms between 4 and 30 days after aSAH | 112 | 404 | 303 | internal verification | 101 | deleted | single-factor + multi-factor tests |
| 12 | Miguel A. Hernández-Hernández [27] | 2022 | Spain | cohort study | single-center study | DCI was diagnosed when a focal neurological impairment or a decrease of at least 2 points on the GCS lasting for at least 1 h occurred not attributable to other causes, or when cerebral infarction on CT or magnetic resonance (MR) scan was seen within 6 weeks after aSAH, not present on the CT or MR scan between 24 and 48 h after early aneurysm occlusion, and not attributable to other causes | 16 | 64 |  |  |  |  | single-factor + multi-factor tests |
| 13 | Alis J Dicpinigaitis [28] | 2022 | USA | cohort study | multi-center study | 1) documentation of symptomatic vasospasm (ICD-9-CM 435.9 or diagnosis string of "vasospasm"), or 2) documentation of new cerebral infarction (ICD-9-CM434.91) (exclusive of iatrogenic and thromboembolic causes) | 33 | 276 |  |  |  |  | single-factor + multi-factor tests |
| 14 | István Csók [29] | 2022 | Germany | cohort study | single-center study | New cerebral infarction on CT or MRI within 6 weeks after aSAH or on the latest CT or MRI before death within 6 weeks, not present on the CT or MRI scan between 24 and 48 h after early aneurysm occlusion and not attributable to other causes such as surgical clipping or endovascular treatment, was classed as DCI | 84 | 417 | 283 | internal verification | 134 |  | single-factor + multi-factor tests |
| 15 | Kehua Chen [30] | 2022 | China | cohort study | single-center study | DCI was diagnosed as clinical deterioration, a new infarction on cerebral CT or MRI scanning, or both. Clinical deterioration caused by DCI was defined as (a) new focal neurological impairment (such as hemiparesis, hemianopia, aphasia, apraxia, or neglect) and (b) a decrease of at least 2 points on the Glasgow Coma Scale (GCS). Clinical deterioration had to last for at least 1 h, was not apparent immediately after accepting aneurysm occlusion, nor be attributed to other causes based on comprehensive assessment (such as relevant laboratory results, CT or MRI scanning of the brain, and clinical assessment). A new infarction on brain CT or MRI was not present on admission or between 24 and 48 h after accepting aneurysm occlusion. Both clinical deterioration and new infarction were assessed by the same two authors independently during hospitalization | 138 | 424 |  |  |  | deleted | single-factor + multi-factor tests |
| 16 | Hsin Yi Chen [31] | 2022 | USA | cohort study | single-center study | (1) new focal neurologic deficits or decrease in the Glasgow Coma Scale of at least 2 points, persisting for a minimum of 1 hour, not explained by other causes (e.g., complications of a procedure, sedation, spike in intracranial pressure, re-rupture, hydrocephalus, systemic or metabolic abnormalities) through clinical assessment, imaging, or laboratory data; or (2) the presence of cerebral infarction on CT or MRI of the brain, acquired at the discretion of the clinical team, that was not present on any neuroimaging done within the first 48 hours following early aneurysm occlusion, and not attributable to other causes such as surgical clipping or endovascular treatment. | 56 | 107 |  | internal verification |  | mean (linear) imputation | single-factor + multi-factor tests |
| 17 | William S. Bolton [32] | 2022 | UK | cohort study | single-center study | occurrence of focal neurological impairment (such as hemiparesis, aphasia, apraxia, hemianopia, or neglect) or a decrease of at least two points on the Glasgow Coma Scale that was not apparent immediately after aneurysm occlusion | 96 | 403 |  |  |  |  | single-factor + multi-factor tests |
| 18 | Georgios Alexopoulos [33] | 2022 | USA | cohort study | single-center study | DCI must meet the criteria for developing new-onset neurological deficits between days 4 and 12 following the aneurysm rupture, defined as worsening of Modified Glasgow coma score for 2 or more points, followed by CT or brain MRI confirmation demonstrating new ischemic infarcts since their admission | 42 | 213 | 149 |  | 64 | imputed via the Multivariate Imputation by Chained Equations (MICE) procedure | multi-factor test |
| 19 | Helena Odenstedt Hergès [34] | 2021 | Sweden | cohort study | single-center study | DCI was defined according to current guidelines as the appearance of focal or global (two-point decrease on GCS scale) neurological impairment and/ or cerebral infarction on CT or MR and not attributed to other causes | 167 | 420 |  | internal verification | 139 | mean value | multi-factor test |
| 20 | Long Zhao [35] | 2021 | China | cohort study | single-center study | detection of any new focal neurological impairment or when there was a minimum 2-point decrease in the Glasgow Coma Scale. The impairment had to last for at least 1 hour, not be apparent immediately after aneurysm occlusion, and not be attributed to other causes through clinical assessment or laboratory tests | 66 | 285 | 214 | internal verification | 71 |  | single-factor + multi-factor tests |
| 21 | Jude P.J. Savarraj [36] | 2021 | USA | cohort study | single-center | DCI is characterized by neurologic worsening occurring between 4 and 21 days after the initial hemorrhage, affecting 20%–30% of patients with SAH | 88 | 399 | 319 | internal verification | 80 | imputed procedurally | multi-factor test |
| 22 | Murad Megjhani [37] | 2021 | USA | cohort study | triple-center study | a ≥2-point change in Glasgow Coma Scale (GCS) or new focal neurological deficit lasting for >1 hour and not associated with surgical treatment or a new cerebral infarct on brain imaging that is not attributable to any other causes. | 124 | 388 | 310 | Multi-center external validation | 38/40 | median | multi-factor test |
| 23 | Masahito Katsuki [38] | 2021 | Japan | cohort study | single-center study | symptomatic vasospasm | 57 | 298 | 248 | internal verification | 50 | automatically compensated | multi-factor test |
| 24 | Ping Hu [39] | 2021 | China | cohort study | double-center study | (1) CT scan omitting other causes of focal neurological decline such as aphasia, apraxia, hemianopia, or neglect, either permanent or temporary, within 4 to 14 days after SAH; (2) a Glasgow Coma Scale decrease of at least 2 points lasting for at least 1 h and not immediately evident after surgery; (3) head CT scan revealing a new cerebral infarction within 4–30 days after aSAH, which was not noticeable on admission or immediately after the operation, and no other explanation except for vasospasm | 57 | 210 | 126 | external verification | 84 |  | multi-factor test |
| 25 | Guido de Jong [40] | 2021 | Netherlands | cohort study | multi-center study | a new focal neurological deficit or decrease in level of consciousness, a new infarct revealed by follow-up CT imaging, or both |  | 362 |  | internal verification |  |  | multi-factor test |
| 26 | Haonan Liu [41] | 2020 | China | cohort study | single-center study | a focal neurological deficit attributable to a detected vascular territory of intracranial arterial narrowing (angiographic vasospasm) in the absence of alternative causes | 224 | 887 | 621 | internal verification | 266 |  | single-factor + multi-factor tests |
| 27 | Satoru Tanioka [42] | 2019 | Japan | cohort study | multi-center study | occurrence of new focal neurological impairments (such as hemiparesis, aphasia, apraxia, and/or neglect), and/or a decrease of at least 2 points on the Glasgow Coma Scale (either on the total score or on one of its components), which lasted for at least 1 h, and were not apparent immediately after aneurysmal obliteration | 13 | 95 |  | internal verification |  |  | multi-factor test |
| 28 | IsabelFragata [43] | 2019 | Portugal | cohort study | single-center study | (1) presenting with a new focal neurological deficit/decrease in the level of consciousness non-attributable to other causes (for example, hydrocephalus, seizures, metabolic derangement, infection, or sedation); (2) presenting a new infarct on follow-up CT /magnetic resonance (MR) imaging; or (3) both 1 and 2, after 4 days after ictus | 15 | 66 |  |  |  |  | single-factor + multi-factor tests |
| 29 | Yuan‐Jian Fang [44] | 2019 | China | cohort study | single-center study | appearing clinical vasospasm or/and delayed cerebral infarction. (a) Clinical deterioration (GCS by ≥2 points, or development of new motor deficits, which excluding other etiologies) was considered as clinical vasospasm; (b) new infarct on brain CT that was not visible on the initial CT, excluding infarctions that appeared around the aneurysm within 48 hours after aneurysm surgery or endovascular treatment, was considered as delayed cerebral infarction | 196 | 702 |  | internal verification | 108 |  | single-factor + multi-factor tests |
| 30 | Marianne L. Bøthun [45] | 2019 | Norway | cohort study | single-center study | a new focal neurological impairment or ≥ 2 points reduction in Glasgow Coma Score, lasting for minimum 1h, and not appearing immediately after aneurysm occlusion. Other causes of deterioration were excluded by clinical assessment, cerebral CT or MRI, and laboratory analyses | 22 | 42 |  |  |  |  | multi-factor test |
| 31 | Fawaz al-Mufti [46] | 2019 | USA | cohort study | single-center study | occurrence of focal neurological impairment, or a decrease in at least two points on the Glasgow coma scale, which was not apparent immediately after aneurysm occlusion, and could not be attributed to other causes | 202 | 1067 |  |  |  |  | single-factor + multi-factor tests |
| 32 | Fawaz Al-Mufti [47] | 2018 | USA | cohort study | single-center study | (1) clinical deterioration (ie, a new focal deficit, decrease in the level of consciousness, or both), and/or (2) a new infarct on CT that was not visible on the admission or immediate postoperative scan, when the cause was thought by the research team to be vasospasm | 196 | 849 |  |  |  |  | single-factor + multi-factor tests |
| 33 | Lucas Alexandre Ramos [48] | 2018 | Netherlands | cohort study | single-center study | defined as the occurrence of new focal neurological impairment or a decrease of two points or more on the Glasgow Coma Scale (GCS) (with or without new hypodensity on CT) that could not be attributed to other causes | 97 | 317 | 238 | internal verification | 79 | imputed using the incremental attribute regression imputation with Random Forest | single-factor + multi-factor tests |
| 34 | Soojin Park [49] | 2018 | USA | cohort study | single-center study | defined as the development of new focal neurological signs or decrease of ≥ 2 points on the Glasgow Coma Scale (GCS), lasting for > 1 h, or the appearance of new infarctions on CT or MRI | 94 | 488 | 390 | internal verification | 98 | zero-padding | multi-factor test |
| 35 | Eric S. Rosenthal [50] | 2018 | USA | cohort study | single-center study | neurological deficit or >=2-point decrement of the Glasgow Coma Scale (GCS) persisting for >=1 hour, not attributable to other causes, including re-rupture, hydrocephalus, elevated intracranial pressure, procedure-related complications, seizures, or systemic or metabolic abnormalities; or infarction on follow-up computed tomography or magnetic resonance imaging | 52 | 103 |  |  |  | carry-forward method | single-factor test |
| 36 | Murad Megjhani [51] | 2018 | USA | cohort study | single-center study | defined as the development of new focal neurologic signs or deterioration of consciousness for >1 h or appearance of new infarctions on imaging due to VSP |  | 488 | 390 | internal verification | 98 | mask decoupling | multi-factor test |
| 37 | Philipp Gölitz [52] | 2017 | Germany | cohort study | single-center study | defined as the occurrence of focal neurological impairment lasting for at least 1 h and not attributable to other causes through clinical assessment, computed tomography (CT) or magnetic resonance imaging (MRI) scanning of the brain, and appropriate laboratory studies | 26 | 52 |  |  |  |  | multi-factor test |
| 38 | Gang Lu [53] | 2017 | China | cohort study | single-center study | a new cerebral infarction identified on computed tomography after exclusion of procedure-related infarctions | 20 | 40 |  | internal verification |  |  | multi-factor test |
| 39 | Isabel Fragata [54] | 2017 | Portugal | cohort study | single-center study | (1) presenting with a new focal neurological deficit/decrease in the level of consciousness nonattributable to other causes (eg, hydrocephalus, seizures, metabolic derangement, infection, sedation), (2) there was a new infarct on follow-up imaging after 4 days post-ictus, or (3) both 1 and 2 | 15 | 60 |  | internal verification |  |  | single-factor + multi-factor tests |
| 40 | Paul M. Foreman [55] | 2016 | USA | cohort study | single-center study | hypoattenuating regions on CT that correspond to a vascular distribution, or an MR image demonstrating a hyperintense area on a diffusion-weighted image sequence with a corresponding hypointense apparent diffusion coefficient sequence corresponding to a vascular territory | 81 | 380 |  | internal + external verification | 255/125 |  | single-factor + multi-factor tests |
| 41 | Ivan Rocha Ferreira Da Silva [56] | 2016 | Brazil | cohort study | single-center study | clinical deterioration deemed secondary to vasospasm (confirmed with digital subtraction angiography) after other causes were eliminated (such as fever, infection, hyponatremia, seizures, hydrocephalus) and radiographic vasospasm as arterial narrowing diagnosed on digital subtraction angiography | 14 | 55 |  |  |  |  | single-factor + multi-factor tests |
| 42 | Fawaz al-Mufti [57] | 2016 | USA | cohort study | single-center study | 1) clinical deterioration, i.e., a new focal deficit, decrease in level of consciousness, or both; and/or 2) a new infarct on CT that was not visible on the admission or immediate postoperative scan, when the cause was believed by the research team to be vasospasm | 296 | 1286 |  |  |  |  | single-factor + multi-factor tests |
| 43 | IJsbrand Zijlstra [58] | 2016 | Netherlands | cohort study | double-center study | clinical DCI, which was defined as clinical deterioration that could not be explained by any cause other than DCI, and radiologic DCI, which was defined as the presence of cerebral infarction on CT or MR images within 6 weeks after SAH or on the latest CT scan or MR image obtained before death, which could not be attributed to other causes such as surgical clipping or endovascular treatment, according to previously published criteria | 68 | 333 |  |  |  |  | single-factor + multi-factor tests |
| 44 | Limin Zhang [59] | 2016 | China | cohort study | single-center study | Probable DCI was defined as a focal deficit or deterioration of the level of consciousness that could not be explained by metabolic disturbances, infection, treatment complications, hydrocephalus, or rebleeding in the absence of new hypodensities on a repeated CT scan. Definite DCI was defined as the deterioration of the level of consciousness, the development of focal neurologic signs, or both, along with evidence of cerebral infarction on CT scan | 223 | 834 |  |  |  |  | single-factor + multi-factor tests |
| 45 | Karol P. Budohoski [60] | 2016 | UK | cohort study | single-center study | DCI was diagnosed on day 8 (range 3–12 days) |  | 98 |  |  |  |  | multi-factor test |
| 46 | Airton Leonardo de Oliveira Manoel [61] | 2015 | Canada | cohort study | multi-center study | The occurrence of focal neurological impairment (such as hemiparesis, aphasia, apraxia, hemianopia, or neglect), or a decrease of at least 2 points on the Glasgow coma scale (either on the total score or on one of its individual components [eye, motor on either side, verbal]). This should last for at least 1 hour, is not apparent immediately after aneurysm occlusion, and cannot be attributed to other causes through clinical assessment, CT or MRI scanning of the brain, and appropriate laboratory studies | 151 | 746 |  |  |  |  | multi-factor test |
| 47 | Nicolien K. de Rooij [62] | 2013 | Netherlands | cohort study | single-center study | decreased Glasgow coma scale of at least 2 points lasting >2 hours or a new focal deficit with exclusion of other causes (rebleed, hydrocephalus, epilepsy, metabolic, or infectious causes) | 162 | 626 | 371 | internal verification | 255 | regression imputation | multi-factor test |
| 48 | Antoine Baumann [63] | 2013 | France | cohort study | single-center study | occurrence of a new delayed neurological deficit with evidence of ischemia on perfusion-diffusion MRI, CT angiography, or CT perfusion imaging, or with cerebral angiography | 16 | 32 |  |  |  |  | multi-factor test |
