# Supplementary material for: Machine Learning for the Early Prediction of Delayed Cerebral Ischemia in Patients With Subarachnoid Hemorrhage: Systematic Review and Meta-Analysis
Source: J Med Internet Res. 2025 Jan 20;27:e54121. doi: 10.2196/54121 (PMC11791451; doi:10.2196/54121)
Supplement: Multimedia Appendix 3 [file jmir_v27i1e54121_app3.docx]

**Multimedia Appendix 3 modeling predictors**

| No | Variable | Frequency |
| --- | --- | --- |
| 1 | Age | 53 |
| 2 | Sex | 46 |
| 3 | Hunt and Hess score | 41 |
| 4 | Modified Fisher scale | 40 |
| 5 | WFNS | 35 |
| 6 | Aneurysm location | 32 |
| 7 | Hypertension (HTN) | 29 |
| 8 | Coiling or clipping | 28 |
| 9 | Smoking/drinking history | 25 |
| 10 | Diabetes | 23 |
| 11 | Laboratory results | 21 |
| 12 | GCS | 16 |
| 13 | Family history of aneurysm | 14 |
| 14 | Coronary heart disease | 12 |
| 15 | Anticoagulant treatment | 11 |
| 16 | Hyperlipidemia | 10 |
| 17 | mRS | 10 |
| 18 | Image CT value | 8 |
| 19 | Intracerebral hemorrhage | 8 |
| 20 | Hydrocephalus | 6 |
| 21 | Brain tissue oxygenation | 5 |
| 22 | Hijdra score | 5 |
| 23 | Length of stay | 5 |
| 24 | VASOGRADE | 5 |
| 25 | Angiographic vasospasm | 3 |
| 26 | Blood pressure | 3 |
| 27 | EVD | 3 |
| 28 | Heart rate | 3 |
| 29 | Respiratory rate | 3 |
| 30 | Apache-II Phys | 2 |
| 31 | CAD | 2 |
| 32 | Cerebral infarction | 2 |
| 33 | Congestive heart failure | 2 |
| 34 | CPP | 2 |
| 35 | Delay between diagnosis and treatment | 2 |
| 36 | Intracranial pressure | 2 |
| 37 | Pressure reactivity index | 2 |
| 38 | Severity on admission | 2 |
| 39 | Cisternal BV | 1 |
| 40 | Early brain injury | 1 |
| 41 | FV | 1 |
| 42 | IVH | 1 |
| 43 | Mechanical ventilation | 1 |
| 44 | miRRNA | 1 |
| 45 | Nine quantitative EEG features | 1 |
| 46 | Qualitative CTP | 1 |
| 47 | Rebleeding | 1 |
| 48 | SEBES | 1 |
| 49 | Sulcal + cisternal BV | 1 |
| 50 | Sulcal BV | 1 |
| 51 | Sxa | 1 |
| 52 | Total blood volume | 1 |
| 53 | TOxa | 1 |
| 54 | Ventricular BV | 1 |

Note: Laboratory results in the table refer to results that are commonly found in medical laboratory departments, which mainly include hematology tests, biochemistry tests, immunology tests, microbiology tests, molecular biology tests, and urine tests. In this study, due to the diversity of laboratory indicators included, we only denoted Laboratory results, which included but were not limited to CRP (mg/L), WBC (×10^9^/L), Neutrophil (×10^9^/L), Lymphocyte (×10^9^/L), Monocyte (×10^9^/L), Eosinophil (×10^9^/L), Procalcitonin (ng/mL), Hematocrit, and High Temperature Requirement A1.
